# Combination of TLR1/2 and TLR3 ligands enhances CD4^+^ T cell longevity and antibody responses by modulating type I IFN production

**DOI:** 10.1038/srep32526

**Published:** 2016-09-01

**Authors:** Bo Ryeong Lee, Soo Kyung Jeong, Byung Cheol Ahn, Byeong-Jae Lee, Sung Jae Shin, Jung Sun Yum, Sang-Jun Ha

**Affiliations:** 1 Department of Biochemistry, College of Life Science & Biotechnology, Yonsei University, Seoul 120749, Republic of Korea; 2 R&D Center, CHA Vaccine Institute, Seongnam-si, Gyeonggi 462716, Republic of Korea; 3 Jeonbuk Department of Inhalation Research, Korea Institute of Toxicology, Jeollabuk-do 56212, Republic of Korea; 4 Department of Microbiology, Institute for Immunology and Immunological Diseases, Brain Korea 21 PLUS Project for Medical Science, Yonsei University College of Medicine, Seoul 120749, Republic of Korea

## Abstract

Despite the possibility of combining Toll-like receptor (TLR) ligands as adjuvants to improve vaccine efficacy, it remains unclear which combinations of TLR ligands are effective or what their underlying mechanisms may be. Here, we investigated the mechanism of action of L-pampo, a proprietary adjuvant composed of TLR1/2 and TLR3 ligands. L-pampo dramatically increased humoral immune responses against the tested target antigens, which was correlated with an increase in follicular helper T cells and the maintenance of antigen-specific CD4^+^ T cells. During the initial priming phase, in contrast to the induction of type I interferon (IFN) and pro-inflammatory cytokines stimulated by polyI:C, L-pampo showed a greatly diminished induction of type I IFN, but not of other cytokines, and remarkably attenuated IRF3 signaling, which appeared to be critical to L-pampo-mediated adjuvanticity. Collectively, our results demonstrate that the adjuvant L-pampo contributes to the promotion of antigen-specific antibodies and CD4^+^ T cell responses via a fine regulation of the TLR1/2 and TLR3 signaling pathways, which may be helpful in the design of improved vaccines.

Toll-like receptor (TLR) ligands are widely studied as adjuvants, particularly in combination with subunit vaccines, because they modulate immune responses, thereby establishing a sufficient amount of protective immunity^1^,^2^. In an effort to identify more efficacious adjuvants, specific combinations of TLR ligands have been found to be better than single-ligand adjuvants^3^,^4^. Some combinations of TLR ligands synergistically enhance both the magnitude and quality of the immune response, including the generation of follicular helper T (Tfh) cells, the survival of antigen-presenting cells (APCs), and the affinity of antibodies^5^,^6^. Indeed, when vaccinated with pathogen-specific antigens, the combinatorial use of TLR ligands was more effective in controlling bacterial and viral infections than single TLR ligands^5^,^6^,^7^. However, the underlying mechanism by which combinations of TLR ligands enhance the immune responses requires further investigation for rational vaccine design.

After vaccination, the maintenance of high frequencies of memory T cells is a critical parameter for maximizing protective efficacy. Upon boosting, pre-existing high frequencies of memory T cells correlate well with memory differentiation, whereas less pre-existing memory cells go through more cell divisions and become senescent^8^. To prevent erosion of the proliferative potential of memory T cells, an extensive mechanistic perspective into the maintenance of memory T cells is necessary. Despite the importance of CD4^+^ T cells in both humoral and cellular protective immunity, factors and adjuvants that control the maintenance of memory CD4^+^ T cells are not well-understood compared to those of memory CD8^+^ T cells^9^,^10^,^11^,^12^,^13^,^14^,^15^.

Here, we investigate how TLR1/2 and TLR3 ligands synergize to enhance antigen-specific T and B cells. We have previously reported that L-pampo, a proprietary adjuvant composed of Pam3Csk4 (Pam3) and polyinosinic:polycytidylic acid (polyI:C), induced a much stronger humoral immune response to surface antigens of hepatitis B virus (HBV) than aluminum hydroxide (alum)^16^. PolyI:C, a synthetic double-stranded RNA (dsRNA), is an agonist of TLR3 and RIG-I that predominantly produces type I interferon (IFN) via the TBK1-IRF3 axis and strongly polarizes T helper 1 (Th1) immunity^17^,^18^. Pam3, a synthetic bacterial lipoprotein, is a TLR1/2 agonist reported to produce pro-inflammatory cytokines such as IL-6 and IL-10 via the NFκB signaling pathway and polarize T helper 2 (Th2) immunity^19^,^20^. When L-pampo is used as an adjuvant in a protein immunization system, it contributes to the maintenance of antigen-specific CD4^+^ T cell responses by regulating the IRF signaling pathway and type I IFN production. The potent L-pampo-adjuvanted CD4^+^ T cell responses, after booster immunization, led to the generation of multifunctional CD4^+^ T cells and class-switched antibodies correlating to the expansion of germinal center B (GC B) cells. Collectively, we propose that L-pampo adjuvanticity significantly modulates the innate cytokine environment and maintains antigen-specific CD4^+^ T cells during the memory phase, which leads to the expansion of functional CD4^+^ T cells, GC B cells and the enhanced production of class-switched antibodies, most likely amplified upon boosting.

## Results

### L-pampo adjuvanticity synergistically enhances antibody production and expands germinal center B cells

**Figure 1:**
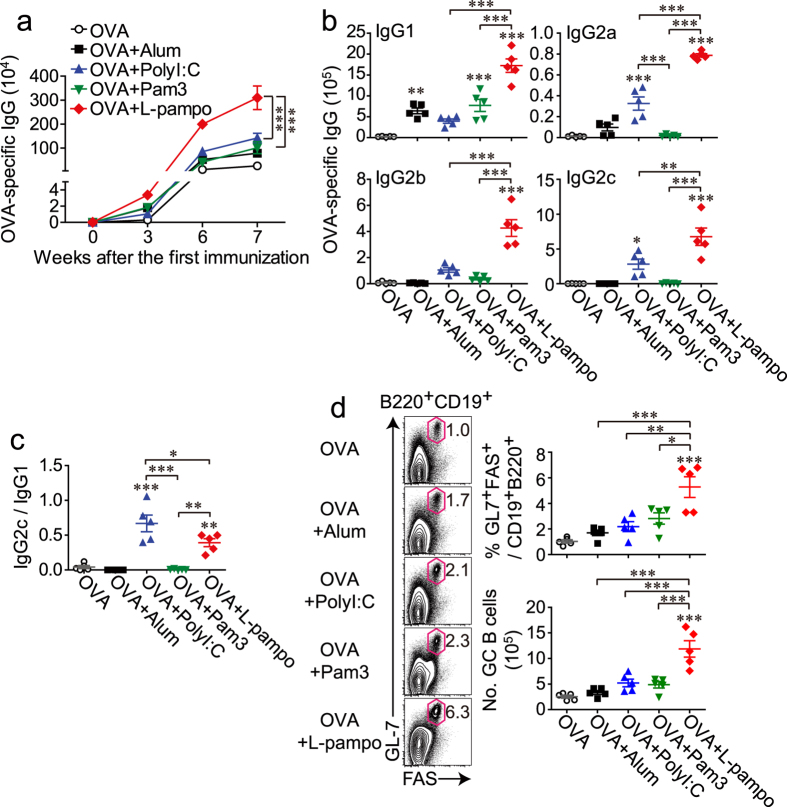
L-pampo is a potent adjuvant that enhances the production of OVA-specific antibodies and expands germinal center B cells upon tertiary immunization with OVA. Naïve B6 mice were immunized with 100 μg of OVA alone or in combination with alum, polyI:C, Pam3, or L-pampo as adjuvants, followed by the same immunization 3 and 6 weeks after the first immunization. (**a**) Individual mouse sera were isolated at the indicated time points after the first immunization, and OVA-specific IgG levels were measured with an ELISA. (**b**) At the 7^th^ week, the indicated isotypes were measured with an ELISA. (**c**) The relative ratios of OVA-specific IgG2c to IgG1 were calculated as an indirect measure of T helper 1 (Th1) and Th2 type immune responses. (**d**) Seven weeks after the first immunization, splenocytes from each mouse were analyzed by flow cytometry. Representative FACS plots and the frequencies and numbers of germinal center B cells (gated as live B220^+^ CD19^+^ GL-7^+^ PNA^+^ lymphocytes) in the spleen of each mouse are shown. Data are represented as the mean ± SEM with each dot indicating one mouse (*n* = 5). The data are representative of two independent experiments. *p < 0.05; **p < 0.01; ***p < 0.001.

To validate the efficacy of L-pampo as an adjuvant, we immunized mice three times at 3-week intervals with ovalbumin protein (OVA) alone or together with polyI:C, Pam3, or L-pampo. Alum was used as a control adjuvant. From the 6^th^ week after the first immunization, a synergistic enhancement of the OVA-specific IgG titer was observed in mice that received L-pampo as an adjuvant (Fig. 1a).

Antibody isotype analysis is one indicator of the type of immune responses^21^. A dominance of IgG1 indicates a Th2-type immunity, whereas a dominance of IgG2a, IgG2b, or IgG2c antibodies implies a Th1-type immunity. As expected, IgG1 was dominant in mice given alum or Pam3, but IgG2 (IgG2a, IgG2b, and IgG2c) was dominant in the polyI:C-treated group. The L-pampo-treated group showed the highest levels of both IgG1 and IgG2 among the experimental groups (Fig. 1b). The ratio of IgG2c to IgG1 in the L-pampo group was higher than that in the alum or Pam3 groups and slightly lower than that in the polyI:C group (Fig. 1c). In conclusion, the combination of polyI:C and Pam3 showed synergistic enhancement of both Th1-type and Th2-type OVA-specific antibodies.

Next, we tested the expansion of germinal center B (GC B) cells, which are closely related to antibody responses. A germinal center is a compartment within a B cell follicle in the secondary lymphoid tissues in which GC B cells go through functional maturation steps, such as somatic hypermutation (SHM) and B cell memory generation, which allow for tight regulation of protective humoral immunity^22^,^23^. Similar to what was observed with antibody production, a synergistic expansion of germinal center B cells was observed in mice that received OVA and L-pampo, indicating a better antibody quality (Fig. 1d).

### L-pampo adjuvanticity modulates the antibody response against the HBV antigen toward a Th1-type response

**Figure 2:**
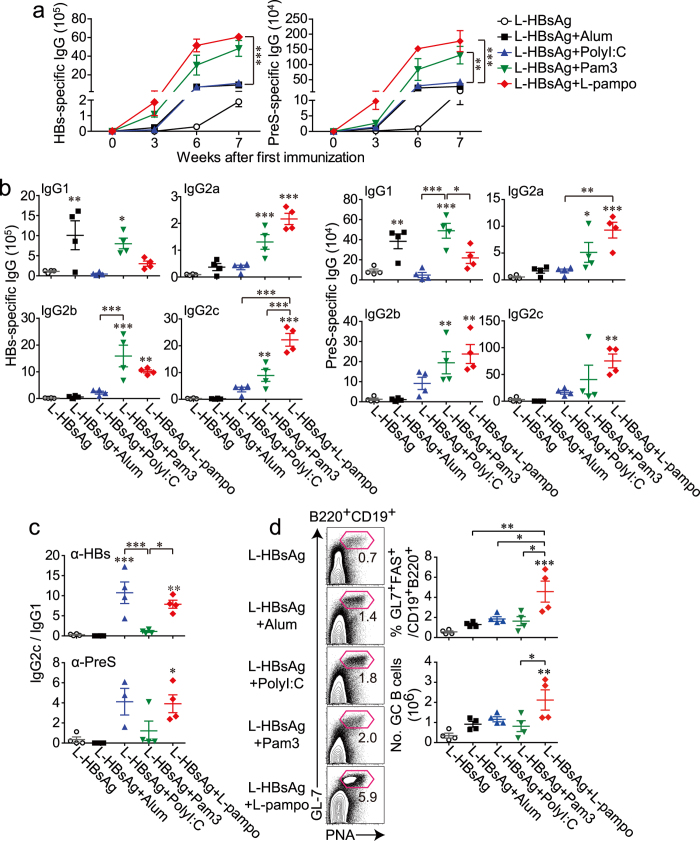
L-pampo is a potent adjuvant that modulates the mode of antibody responses and expands germinal center B cells upon tertiary immunization with the HBV surface protein. Naïve B6 mice were immunized with 40 μg of L-HBsAg, an HBV surface protein, alone or in combination with alum, polyI:C, Pam3, or L-pampo as adjuvants, followed by the same immunization 3 and 6 weeks after the first immunization. (**a**) Individual mouse sera were isolated at the indicated time points after the first immunization, and HBs- or PreS-specific IgG was measured by an ELISA. (**b**) At 7 weeks, the indicated isotypes were measured by an ELISA. (**c**) The relative ratios of HBs- or PreS-specific IgG2c to IgG1 were calculated. (**d**) Seven weeks after the first immunization, splenocytes were analyzed by flow cytometry. Representative FACS plots and the frequencies and numbers of germinal center B cells (gated as live B220^+^ CD19^+^ GL-7^+^ PNA^+^ lymphocytes) in the spleen are shown. Data are represented as the mean ± SEM with each dot indicating one mouse (*n* = 4). *p < 0.05; **p < 0.01; ***p < 0.001.

To examine whether the adjuvant effect of L-pampo would also apply to a different protein antigen, L-HBsAg, which includes HBV surface antigens S and preS proteins, was used with the same series of adjuvants as described above. Similar to the increased OVA-specific antibody response by L-pampo, L-HBsAg-specific IgG production was the highest in the mice given the L-pampo adjuvant (Fig. 2a). In this system, Pam3 strongly induced a substantial production of HBs-specific or PreS-specific antibodies with a relatively low IgG2c/IgG1 ratio, whereas polyI:C did not induce antibody titers as well as Pam3 but still had a higher IgG2c/IgG1 ratio than Pam3 (Fig. 2a–c). When L-HBsAg was combined with L-pampo, the ratio of IgG2c/IgG1 was as high as it was when combined with polyI:C (Fig. 2c). The induction of high titers of Th1-polarized antibodies against L-HBsAg would be beneficial because Th1-type isotypes are closely related to cellular immunity, which is critical to defense against cell-residing viruses.

Interestingly, a synergistic expansion of GC B cells was observed in the L-pampo-adjuvanted group, which implies that L-pampo adjuvanticity most likely induces a better functional antibody response, such as the production of antibodies with higher affinity, despite the similar antibody titers observed in Pam3 adjuvanticity (Fig. 2d).

### Pam3 and L-pampo promote the expansion of Tfh cells in the periphery, but only L-pampo adjuvanticity prominently maintains OVA-specific CD4^+^ T cells during the memory phase

**Figure 3:**
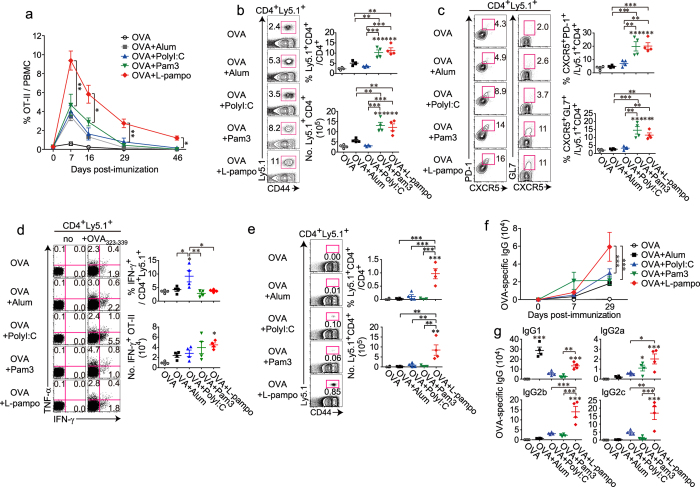
Pam3 and L-pampo promote the expansion of follicular helper T cells in the periphery, but only L-pampo adjuvanticity prominently maintains OVA-specific CD4^+^ T cells during the memory phase. Naïve Ly5.2^+^ B6/J mice were injected with Ly5.1^+^ OT-II cells (5 × 10^5^ cells each) before the immunization and immunized with 100 μg of OVA alone or in combination with alum, polyI:C, Pam3, or L-pampo as adjuvants. (**a**) Mouse PBMCs were isolated from the blood, and the frequency of the transferred OT-II cells (CD4^+^ CD44^+^ Ly5.1^+^) among CD4^+^ T cells was analyzed kinetically by flow cytometry at the indicated time points after immunization. (**b–d**) Eight days after the immunization, splenocytes were isolated and analyzed by flow cytometry. (**b**) Representative FACS plots and the frequencies and numbers of the transferred OT-II cells among CD4^+^ T cells (gated as CD4^+^ CD44^+^ Ly5.1^+^). (**c**) Representative FACS plots and the frequencies of PD-1^+^ CXCR5^+^ follicular helper T cells (Tfh) or GL-7^+^ CXCR5^+^ germinal center Tfh (GC-Tfh). (**d**) Concatenated FACS plots of cytokine-producing OT-II cells and the frequencies and numbers of IFN-γ^+^ cells among the OT-II cells. (**e**) Forty-eight days after immunization, splenocytes were isolated and analyzed by flow cytometry. Representative FACS plots and the frequencies and numbers of the transferred OT-II (CD4^+^ CD44^+^ Ly5.1^+^) cells among CD4^+^ T cells. (**f,g**) Mouse serum was isolated at the indicated time points, and OVA-specific IgG (**f**) and the indicated isotypes at day 29 (**g**) were determined by an ELISA. The data are represented as the mean ± SEM with each dot indicating one mouse (*n* = 3–4). The data are representative of two independent experiments. *p < 0.05; **p < 0.01; ***p < 0.001.

To dissect the underlying mechanism for the enhanced antibody responses by L-pampo, we analyzed OVA-specific CD4^+^ T cell responses over time, including the effector and memory phases. To monitor the responses, OT-II cells were adoptively transferred prior to OVA immunization. OT-II cells in the blood of each adjuvant group expanded well at the effector phase and gradually contracted until day 46 after immunization (Fig. 3a). However, OT-II cells from the L-pampo group expanded the most and remained in the blood at significantly high levels even at day 46 and in the spleen at day 48 (Fig. 3a,e).

To examine antigen-specific CD4^+^ T cell and B cell responses more closely, the lymphocytes in the spleens were analyzed at day 8 post-immunization. Tfh cells are a specialized subtype of CD4^+^ T cells used for B cell help; therefore, they are critical for enhancing antibody responses^24^. CXCR5 expression is a signature that enables Tfh to localize in the B cell zone and form germinal centers by interacting with cognate B cells^25^. A considerable expansion of PD-1^+^ CXCR5^+^ Tfh cells and GL7^+^ CXCR5^+^ germinal center-Tfh (GC-Tfh) cells was observed in mice immunized with OVA plus Pam3 or L-pampo (Fig. 3b,c). With regard to Th1 differentiation, OT-II cells from the polyI:C group had the highest frequency of IFN-γ^+^ cells, but the number of IFN-γ^+^ OT-II cells was generally similar among each adjuvant group (Fig. 3d). Taken together, our results indicate that both L-pampo and Pam3 adjuvanticity expands and polarizes more Tfh cells than the other adjuvants at the effector phase, but L-pampo is the only adjuvant that significantly supports the longevity of OVA-specific CD4^+^ T cells.

At day 29 post-immunization, the highest titers of OVA-specific antibodies and isotypes, particularly the Th1-type, were observed in the mouse serum from the L-pampo group (Fig. 3f,g). This could be related to the high number of OT-II cells in the L-pampo group. That is, OVA-specific CD4^+^ T cells from the L-pampo group were significantly maintained and thus likely supported antibody responses well.

### L-pampo attenuates type I IFN production and IRF signaling induced by polyI:C but amplifies AP-1/NFκB signaling

**Figure 4:**
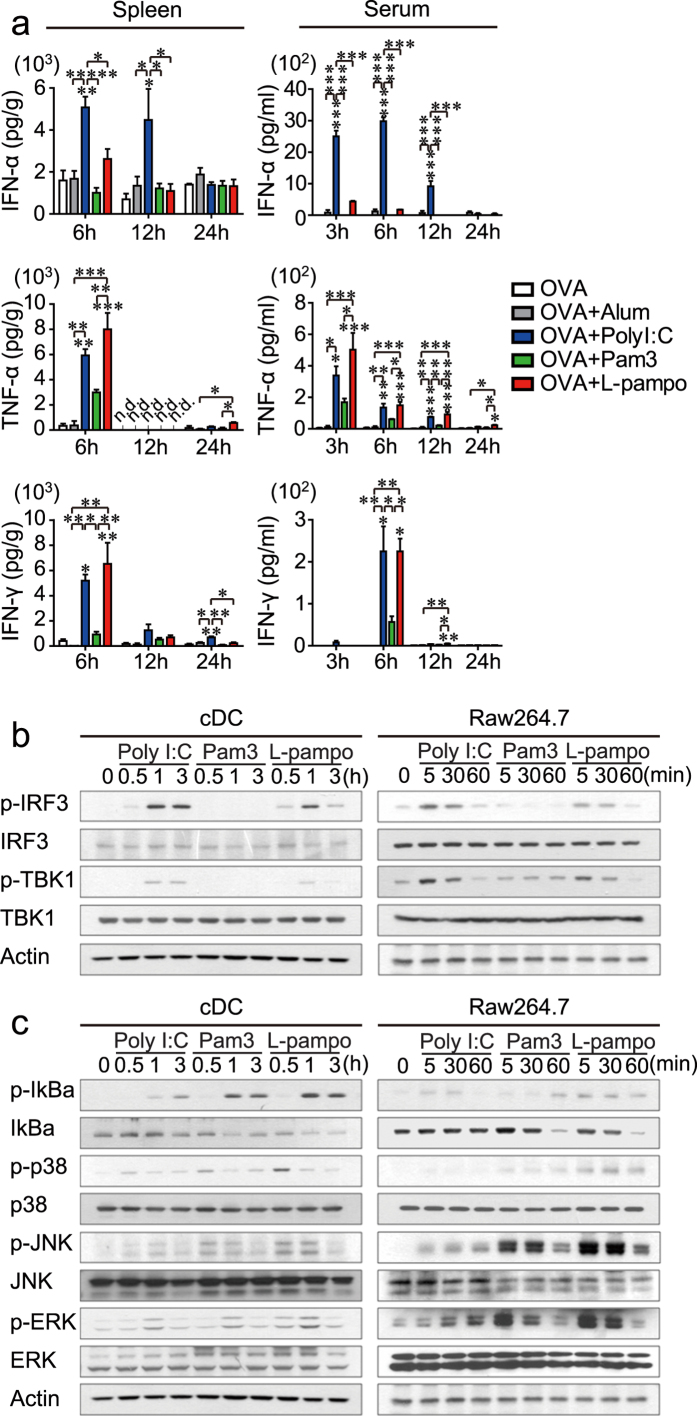
L-pampo attenuates type I IFN secretion *in vivo* and polyI:C-induced IRF3 signaling. (**a**) Naïve B6 mice were immunized with 100 μg of OVA alone or in combination with alum, polyI:C, Pam3, or L-pampo as adjuvants. The spleen and serum of each immunized mouse were collected at the indicated time points, and the levels of IFN-γ, TNF-α, and IFN-α were determined using a multiplex analysis. The level of cytokines detected in the spleen supernatant was divided by the weight (g) of the spleen of that mouse. n.d., not detected. The data are represented as the mean ± SEM (*n* = 3); *p < 0.05; **p < 0.01; ***p < 0.001. (**b**,**c**) CD11c^+^ conventional dendritic cells (cDCs) isolated from flt3L-expressing-B16F10-injected mice (left column) or cells from the mouse macrophage cell line Raw264.7 (right column) were treated with polyI:C, Pam3, or L-pampo for the indicated time; western blots were performed to quantify proteins in the TBK1-IRF3 signaling pathway (**b**) or the AP-1/NFκB signaling pathway (**c**). The data are representative of two independent experiments.

To investigate the factors that support the differentiation and maintenance of CD4^+^ T cells, we analyzed the effect of L-pampo on the *in vivo* profiles of secreted cytokines and signaling molecules in the TLR pathway in the first 24 hours, which could be a critical time point for the onset of T cell differentiation. As expected, polyI:C produced a substantial amount of IFN-α and IFN-γ, whereas Pam3 produced little IFN-α and IFN-γ but a significant amount of pro-inflammatory cytokines such as TNF-α in both the serum and the spleen (Fig. 4a). Surprisingly, when polyI:C and Pam3 were combined (L-pampo), a significantly attenuated level of IFN-α but a similar amount of IFN-γ and TNF-α were observed in the serum and spleen compared to the amounts in the polyI:C group (Fig. 4a).

To verify the L-pampo-mediated attenuation of type I IFN production in TLR signaling molecules, CD11c^+^ conventional dendritic cells (cDCs) enriched *in vivo* were isolated and treated *ex vivo* with polyI:C, Pam3, or L-pampo for 0.5, 1, or 3 h. The up-regulation of phosphorylated TBK1 and IRF3 observed in polyI:C-treated cDCs was significantly attenuated after 3 h of stimulation with L-pampo, reaching levels observed in the attenuated IFN-α production above (Fig. 4a,b). Likewise, a similar effect was observed in a macrophage cell line, Raw264.7, confirming that the activation of the TBK1-IRF3-type I IFN axis by polyI:C is considerably attenuated by Pam3 in L-pampo-treated Raw264.7 (Fig. 4c). However, signaling molecules such as phosphorylated JNK, IκB, and p38 that convergent to activate NFκB or AP-1 were generally amplified in both cDCs and Raw264.7 cells when polyI:C and Pam3 were combined as L-pampo (Fig. 4c). Thus, L-pampo modulates TLR signaling pathways triggered by polyI:C or Pam3 alone, thereby leading to a different innate cytokine environment in which the production of type I IFNs is selectively attenuated.

It has been well understood that adjuvants promote the maturation of APCs by up-regulating co-stimulatory molecules such as CD40, CD80, or CD86 together with major histocompatibility complex (MHC) molecules, leading to a better priming of T cells^3^,^26^. In this regard, we analyzed the subtype-specific maturation status of DCs by profiling the expression of co-stimulatory molecules and MHC molecules during the first 24 h after immunization (Fig. S1a). Indeed, the co-injection of polyI:C, Pam3, or L-pampo along with OVA induced a more potent upregulation of CD86 in lymphoid and myeloid DCs than an OVA immunization alone. The expressions of CD80, CD40, and MHC class II molecules were also slightly enhanced in lymphoid DCs in adjuvant-treated groups (Fig. S1b). However, no enhanced expression of such molecules was observed in DCs in the L-pampo group compared to the polyI:C or Pam3 group (Fig. S1b).

### L-pampo has the ability to generate a high quantity and quality of memory CD4^+^ T cells

**Figure 5:**
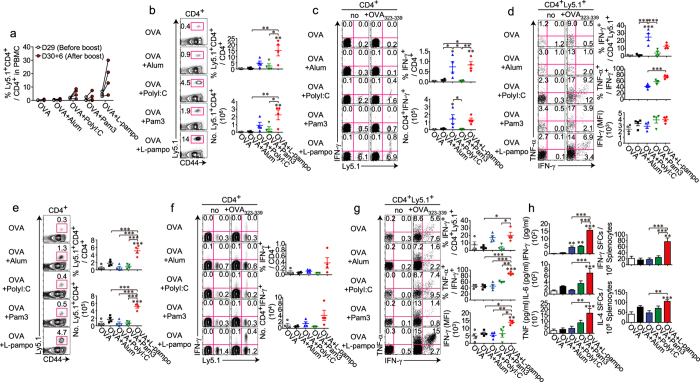
L-pampo expands multi-cytokine-producing antigen-specific CD4^+^ T cells more synergistically as the number of boosts increases. Naïve Ly5.2^+^ B6/J mice were injected with Ly5.1^+^ OT-II T cells (5 × 10^5^ each) before the immunization, then immunized with 100 μg of OVA alone or in combination with alum, polyI:C, Pam3, or L-pampo as adjuvants, followed by the same immunization at day 30 (**a**–**d**) or 3 or 6 weeks after the first immunization (**e**–**h**). (A) Mouse PBMCs were isolated from the blood, and the frequencies of the transferred OT-II cells (CD4^+^ CD44^+^ Ly5.1^+^) among CD4^+^ T cells were analyzed kinetically by flow cytometry at the indicated time points. (**b**–**h**) Six days after the secondary immunization (**b**–**d**) or 7 days after the tertiary immunization (**e**–**h**), splenocytes were isolated and analyzed by flow cytometry. (**b**,**e**) Representative FACS plots and the frequencies and numbers of the transferred OT-II cells (CD4^+^ CD44^+^ Ly5.1^+^) among CD4^+^ T cells are shown. (**c**,**f**) Representative (**c**) or concatenated (**f**) FACS plots and the frequencies and numbers of IFN-γ^+^-producing CD4^+^ T cells among total CD4^+^ T cells are shown. (**d**,**g**) Representative (**d**) or concatenated (**g**) FACS plots of cytokine-producing OT-II cells and the frequencies of IFN-γ^+^ cells among the OT-II cells and of IFN-γ^+^ TNF-α^+^ cells among IFN-γ^+^ OT-II cells and the IFN-γ expression levels of IFN-γ^+^ OT-II cells were determined using the mean fluorescence intensity (MFI) of IFN-γ. (**h**) Splenocytes were stimulated *ex vivo* with OVA_323–339_ peptides. The levels of IFN-γ, IL-6, and TNF in the supernatants of the re-stimulated splenocytes were measured by a cytokine ELISA (left column). Splenocytes were analyzed for IFN-γ or IL-4 secretion by an ELISpot assay (right column). The data are represented as the mean ± SEM with each dot indicating one mouse (*n* = 4). *p < 0.05; **p < 0.01; ***p < 0.001.

To analyze the boosting efficacy of each adjuvant, the expansion and cytokine-producing abilities of OVA-specific CD4^+^ T cells were examined after the boost. Notably, the expansion of OT-II cells in the blood was the most prominent in the L-pampo group (Fig. 5a). Compared to the other groups, the L-pampo group had the highest frequency and the most abundant numbers of OT-II cells in the spleen after the boost (Fig. 5b). Moreover, although the levels of IFN-γ produced (% IFN-γ^+^ among OT-II cells) in the L-pampo group were comparable to those produced in the polyI:C group, the percentage of multi-cytokine-producing OT-II cells (TNF-α^+^ IFN-γ^+^ among IFN-γ^+^ cells) was significantly higher than in the other groups (Fig. 5c,d).

The boost efficacy was far more amplified by the second boost. The spleens of the recipient mice were analyzed one week after the third immunization. OT-II cells from the L-pampo group expanded synergistically compared to those from the polyI:C or Pam3 groups (Fig. 5e). Moreover, the IFN-γ-producing capability of OT-II cells was higher in the L-pampo group than in the other groups, and their multi-cytokine-producing ability (TNF-α^+^ IFN-γ^+^ among IFN-γ^+^ cells) was also superior to that observed in the other groups (Fig. 5f,g). Considering the number of OT-II cells in the L-pampo group and their ability to produce multi-cytokines together, the overall production of the cytokines should be much higher. This was verified by a cytokine ELISA of IFN-γ, IL-6, and TNF secreted from splenocytes and by an ELISpot assay of IFN-γ- and IL-4-secreting cells among splenocytes, which were *ex vivo* stimulated with OVA_323–339_ peptides (Fig. 5h) or OVA (Fig. S2a,b).

The level of expansion of the transferred OVA-specific CD8^+^ T cells (OT-I cells) from the L-pampo group was a little lower than that of the polyI:C group (Fig. S3a), but their functional capability to produce IFN-γ and multi-cytokines was superior to the other groups, as determined by the frequency of TNF-α^+^ IFN-γ^+^ among IFN-γ^+^ cells and their intensity of IFN-γ expression after *ex vivo* stimulation (Fig. S3b). The amount of IFN-γ and TNF-α secreted from the splenocytes and the spot numbers of IFN-γ-secreting splenocytes, when stimulated with the OVA_257–264_ peptide, appeared to be similar between the polyI:C and L-pampo groups, but IL-6 secretion was higher than in the L-pampo group than in the other groups (Fig. S3c,d). Therefore, L-pampo adjuvanticity appears to enhance the expansion of multi-functional antigen-specific T cells, particularly CD4^+^ T cells, compared to polyI:C or Pam3 administered alone.

## Discussion

In this study, we report an efficacious combination of TLR ligands that synergistically enhances the antibody responses against two different protein antigens. As a mechanism of action, we propose that the combination of polyI:C and Pam3 results in a cytokine environment that is distinct from the environment resulting from the use of polyI:C or Pam3 alone, exhibiting a prominent maintenance of antigen-specific CD4^+^ T cells during the memory phase. The expansion of functional CD4^+^ T cells is likely amplified during each round of boosting, leading to the significant expansion of GC B cells and the production of isotype-switched antibodies in mice immunized with OVA and L-pampo.

First, we examined the changes in a signaling pathway resulting from the combination of Pam3 and polyI:C. PolyI:C and Pam3 both inhibit and synergize together to form a unique cytokine environment. When polyI:C and Pam3 are combined, the robust phosphorylation of TBK1 and IRF3 by polyI:C was inhibited by Pam3, whereas that of NFκB and AP-1 signaling molecules was synergistically enhanced, both in primary CD11c^+^ cells and in a macrophage cell line. These findings are consistent with a previous report that showed that the levels of some interferon-stimulated genes (ISGs) and *ifn-b* mRNA were reduced when mouse bone marrow-derived dendritic cells (BMDCs) were treated with polyI:C and Pam3^27^. Mechanistically, the polyI:C-mediated induction of type I IFNs has been reported to be directly inhibited by TLR1/2 adaptor proteins such as MyD88 and MyD88 adaptor-like (Mal) or negatively regulated by the NFκB subunits RelB and cRel via transcriptional repressor protein YY1 in bone marrow-derived macrophages (BMDMs) and BMDCs^28^,^29^,^30^,^31^.

At the effector phase, we showed that the expansion of Tfh was higher in L-pampo- and Pam3-treated groups. Recently, it was shown that type I IFN acts as a repressor of signal transducer and activator of transcription 3 (STAT3), thereby limiting Tfh differentiation in a virus infection model^32^. This report partially provides a link between the attenuated type I IFN production and expansion of Tfh. Because of the importance of Tfh in forming and supporting the GC response, the expansion of Tfh by L-pampo and Pam3 is thought to contribute to the enhancement of antibody titers and isotype-switching later.

Interestingly, what makes L-pampo different from Pam3 and other adjuvants is its ability to adequately maintain antigen-specific CD4^+^ T cells during the memory phase. Because factors affecting the maintenance of antigen-specific CD4^+^ T cells are relatively under-studied compared to those for CD8^+^ T cells, many questions remain as to whether the maintenance of antigen-specific CD4^+^ T cells is programmed when they are primed at the early phase, sustained by a putative continuous signal during the late phase, or both. If the former is true, the early cytokine environment might have a role in programming maintenance. In our experimental system, different profiles of type I IFN and IFN-γ secretion were observed among the groups during the early phase. In fact, the role of type I IFN and IFN-γ in CD4^+^ T cell homeostasis is controversial depending on the conditions. A recent review describes important roles of type I IFN for the survival of CD4^+^ T cells depending on the context of TCR stimulation and the mode of infection^33^. A direct requirement of type I IFN for the survival of CD4^+^ T cells is not observed in bacterial immunization^34^. Similarly, direct IFN-γ signaling is reported to enhance CD4^+^ T cell memory in a virus infection model^35^, whereas the opposite effect is reported for CD8^+^ T cells in a peptide immunization model^36^. Therefore, further studies are required to determine the complex effects of type I IFN and IFN-γ on the expansion, differentiation, and maintenance of CD4^+^ T cells.

Upon several rounds of boosting, multi-functional antigen-specific CD4^+^ T cells that were thought to be amplified and further matured from the remaining memory CD4^+^ T cells were more abundant in mice immunized with OVA and L-pampo compared to the other groups. The multi-cytokine-producing CD4^+^ T cells are critical for clearing or reducing low levels of viruses in response to infection with various types of viruses, including HIV, influenza virus, Epstein-Barr virus (EBV), varicella-zoster virus (VZV), or cytomegalovirus (CMV), and correlating protection against a second challenge with *L. major*^37^. Furthermore, a higher quality of CD8^+^ T cells producing multi-cytokines was observed in the L-pampo group compared to those in the other groups despite a similar number of antigen-specific CD8^+^ T cells in the polyI:C group. Therefore, L-pampo enhances the quality of antigen-specific T cells upon boosting.

When L-pampo is used for clinical application, the toxicity issue should be carefully considered as a high-dose of pathogen-associated molecular patterns (PAMPs) could induce cytokinemia. Indeed, it is reported that TLR3 mediates the acute inflammation of skin after exposure to ultraviolet B (UVB) radiation^38^. Recently, phosphorothioate ODN-guided dsRNA (sODN-dsRNA), which triggers selectively TLR3 with no ability to activate RIG-I/MDA5, is reported to be less toxic because of its capability to induce less pro-inflammatory cytokines but mount comparable anti-tumor cellular immunity compared to polyI:C that triggers both TLR3 and RIG-I/MDA5 pathways^39^. If we need a higher dose of polyI:C which is efficacious but could be toxic, TLR3-selective ligand seems to be a good alternative to reduce the toxicity.

Similar to our observation on the synergy between TLR ligands as an adjuvant, some studies have also shown that combinations of TLR ligands can provide advantages as vaccine adjuvants. The combinations provided more protection against pathogens than the constituents given individually. When rhesus monkeys were vaccinated with inactivated pandemic influenza virus mixed with MPL (TLR4 ligand) and R837 (TLR7 ligand), a robust humoral immune response was synergistically induced^5^. In this system, adjuvants activating three TLR ligands via MPL and R848 (TLR7 and TLR8 ligands, respectively) induced a greater antibody response, even greater than what is required for protection after a boost^5^. Indeed, the triple combination of polyI:C, MALP2 (TLR2/6 ligand), and CpG (TLR9 ligand) vaccinated with the HIV envelope peptide showed a remarkable protection against HIV envelope protein-expressing VV compared to that observed for the dual combinations^6^. In this context, our study provides an efficacious TLR ligand combination, composed of the TLR1/2 and TLR3 ligands, that synergistically enhances the quality of antibodies and T cells as well as the quantity of antibodies and CD4^+^ T cells, which have implications for efficacious vaccine development.

## Methods

### Mice

C57BL/6 (000664), CD45.1 (002014), OT-II (004194), and OT-I (003831) mice were purchased from the Jackson Laboratory. For the identification of transferred cells within hosts, OT-II and OT-I mice were bred onto homo- or heterozygous CD45.1 allele backgrounds. All of the animal experiments were conducted in accordance with the Korean Food and Drug Administration guidelines. Protocols were reviewed and approved by the Institutional Animal Care and Use Committee (IACUC) of the Yonsei Laboratory Animal Research Center (YLARC) at Yonsei University.

### Adoptive cell transfers

Congenically marked (CD45.1) splenic CD4^+^ T cells specific to the OVA_323–339_ epitope of OVA from naive OT-II transgenic mice were isolated using the Magnisort^TM^ Mouse CD4 T Cell Enrichment Kit (eBioscience), and 5 × 10^5^ of the isolated OT-II cells were transferred intravenously into naïve B6 (CD45.2) mice before immunization. In some experiments, splenic CD8^+^ T cells specific to the OVA_257–264_ epitope of OVA from naïve OT-I transgenic mice were isolated separately alongside the OT-II cells, and 5 × 10^5^ of the isolated OT-I/OT-II cells from each mouse (total 1 × 10^6^/mouse) were intravenously co-transferred into naïve B6 (CD45.2) mice before immunization.

### Immunization

The preparation of L-HBsAg and the formulation of L-pampo (Korean patent 10-0900837), which is a mixture of pam3csk and polyI:C, was conducted as previously described^16^. Briefly, the entire coding region of the HBV envelope gene (pre-S1-pre-S2-S) was ligated into the pMSG vector (Korean patent 10-2000-0043996) and transfected into CHO cells to obtain high amounts of L-HBsAg, including L, M, and S proteins. L-HBsAg was purified from the culture medium of the recombinant CHO cell line that showed the highest expression level of L-HBsAg^16^.

Each B6 mouse was immunized intraperitoneally with 100 μg of OVA or 40 μg of L-HBsAg, in combination with 500 μg of alum (Alhydrogel; Brenntag Biosector), 125 μg of pam3csk (C-tri), 100 μg of polyI:C (Yamasa), or L-pampo as adjuvants in 400 μl of buffer containing 150 mM NaCl and 10 mM sodium phosphate (pH 6.9).

### *In vivo* cytokine secretion

After immunization, the supernatants from homogenized spleens and serum were collected at the indicated time points. A multiplex analysis of IFN-γ, IFN-α, TNF-α, and IL-6 was assayed using Flowcytomix (eBioscience) according to the manufacturer’s instructions. The samples were read using a FACS Canto II (BD) and analyzed using Flowcytomix Pro software (eBioscience).

### Flow cytometry

Single-cell suspensions were prepared by gentle mechanical disruption of spleens, followed by lysis with ACK lysis buffer to remove RBCs, then surface-stained with anti-CD4 (RM4-5), CD8 (53–6.7), CD45.1 (A20), CD44 (IM7), PD-1 (RMP1-30), B220 (RA3-6B2), CD19 (ebio1D3), CD95 (Jo2), or T cell– and B cell–activation antigen (GL7) antibodies (directly conjugated by FITC, PE, PerCP-Cy5.5, PE-Cy7, APC, BV421, or BV605, respectively). Dead cells were excluded by using LIVE/DEAD Fixable Violet Dead Cell Stain (Life Technologies). A three-step CXCR5 staining was performed as previously described using purified rat anti-mouse CXCR5 (BD), a secondary biotin-conjugated Affinipure Goat anti-rat IgG (Jackson ImmunoResearch), and finally with streptavidin-APC (Invitrogen) or streptavidin-BV605 (Bioclone)^40^. For Bcl6 (K112-91) staining, the cells were stained for surface antigens, followed by permeabilization, fixation, and staining using the Foxp3 Permeabilization/Fixation Kit and Protocol (eBioscience). Intracellular cytokine staining of IFN-γ (XMG1.2), TNF-α (MP6-XT22), and IL-2 (JES6-5H4) was performed using the Cytofix/Cytoperm Kit (BD Pharmingen) according to the manufacturer’s instruction. All of the staining antibodies were purchased from BD, eBioscience, or BioLegend. Flow cytometric data were collected on a FACS Canto II (BD) and analyzed using the FlowJo software (TreeStar).

### *In vivo* DC generation and cell isolation

Splenic dendritic cells were expanded in the mice as previously described^41^. Briefly, B6 mice were injected subcutaneously with 5 × 10^5^ Flt3L-secreting B16F10 melanocytes in Hank’s Balanced Saline Solution (HBSS). After 2 weeks, the spleens were collected, digested by collagenase, and filtered through a mesh before magnetic isolation. Single-cell suspensions of the prepared splenocytes were pre-enriched by magnetic depletion of B and T cells with microbead-conjugated antibodies to CD19 (Miltenyi Biotec) and CD90.2 (Miltenyi Biotec), respectively. The depleted cells were then subjected to magnetic selection of CD11c^+^ conventional dendritic cells using CD11c microbeads (Miltenyi Biotec) to >90% purity.

### Cell culture

The mouse macrophage cells and RAW 264.7 cells were cultured in high glucose Dulbecco’s Modified Eagle’s medium (DMEM; Welgene) supplemented with 5% fetal bovine serum and 1% antibiotics. The cell line was maintained at 37 °C under an atmosphere of 5% CO_2_.

### Cytokine ELISA assay

Splenocytes (1.5 × 10^6^cells/100 μl) from immunized mice were stimulated with antigen (OVA (100 μg/ml) or L-HBs (40 μg/ml)) or OVA_323–339_ peptide and OVA_257–264_ peptides for 24–48 h, and non-stimulated splenocytes were used as a negative control. After stimulation, the cytokines in the supernatants were measured by an ELISA assay according to the manufacturer’s protocol (BD).

### Enzyme-linked immunospot (ELISpot) assay

The mouse IFN-γ/IL-4 ELISPOT assay (Mabtech) was performed according to the manufacturer’s protocol. Briefly, splenocytes (1 × 10^6^ cells/100 μl) were added to a 96-well ELISPOT plate pre-coated with anti-mouse monoclonal IFN-γ or IL-4 antibodies. The cells were incubated with antigens (OVA (100 μg/ml), L-HBs (40 μg/ml)), OVA_323–339_ peptides, and OVA_257–264_ peptides or medium alone for 24–48 h. After stimulation, the wells were washed and incubated with biotinylated anti-mouse IFN-γ or IL-4 antibody for 2 h at RT. Subsequently, the wells were washed and incubated with streptavidin-horseradish peroxidase (HRP) for 1 h. Spot-forming cells (SFC)s were developed with TMB substrate and counted using the AELVIS ELISPOT Reader.

### Western blot analysis

The cells were lysed in RIPA buffer (50 mM Tris-HCl, 150 mM NaCl, 1% Triton X-100, 0.5% sodium deoxycholate and 0.1% SDS) supplemented with protease and phosphatase inhibitors for 30 min on ice. The cell lysates were cleared by centrifugation. The proteins were resolved by SDS-PAGE and transferred to a nitrocellulose membrane (Whatman/GE). The membranes were probed with antibodies. The following were used for western blotting: antiphospho-IRF3 (Cell Signaling), anti-IRF3 (Cell Signaling), antiphospho-IRF7 (Cell Signaling), anti-IRF7 (Cell Signaling), antiphospho-TBK1 (Cell Signaling), anti-TBK1 (Santa Cruz), antiphospho-IkBa (Cell Signaling), anti-IkBa (Cell Signaling), antiphospho-p38 (Cell Signaling), anti-p38 (Cell Signaling), antiphospho-JNK (Santa Cruz), anti-JNK (Santa Cruz), antiphospho-ERK (Santa Cruz), and anti-actin (Santa Cruz). Visualization was performed on film using ECL Plus (GE Healthcare).

### Antibody titer analysis

Mouse serum was obtained from the mouse eyes. The antibody titers were determined using a standard ELISA protocol. Briefly, 96-well microplates were coated with Ag (OVA or L-HBs) at a concentration of 1 μg/ml and blocked with bovine serum albumin (1%) for 1 h. The plates were incubated in a 37 °C incubator. After the microplates were washed, diluted serum was added to each well, and the plates were incubated at 37 °C for 2 h. Then, HRP-conjugated anti-mouse IgG (KPL) was added as a secondary antibody, and the plates were incubated at 37 °C for 1 h. After washing, a color-developing agent (tetramethylbenzidine [TMB]-peroxidase solution; KPL) was added, and the mixture was incubated at room temperature for 5 min. Then, the optical density (OD) of each well was measured at 450 nm using an automatic ELISA plate reader. To determine the titers of the antibody isotypes, a mouse monoclonal antibody isotyping reagent was used for IgG1, IgG2a, IgG2b (Sigma), and IgG2c (SouthernBiotech).

## Additional Information

**How to cite this article**: Lee, B. R. *et al.* Combination of TLR1/2 and TLR3 ligands enhances CD4^+^ T cell longevity and antibody responses by modulating type I IFN production. *Sci. Rep.* **6**, 32526; doi: 10.1038/srep32526 (2016).

## Supplementary Material

Supplementary Information
