# Supplementary material for: Combination of TLR1/2 and TLR3 ligands enhances CD4+ T cell longevity and antibody responses by modulating type I IFN production
Source: Sci Rep. 2016 Sep 1;6:32526. doi: 10.1038/srep32526 (PMC5007540; doi:10.1038/srep32526)
Supplement: Supplementary Information [file srep32526-s1.pdf]

SUPPORTING INFORMATION

Combination of TLR1/2 and TLR3 ligands enhances CD4$^+$ T cell longevity and antibody responses by modulating type I IFN production

Bo Ryeong Lee[1], Soo Kyung Jeong[2], Byung Cheol Ahn[2], Byeong-Jae Lee[3], Sung Jae Shin[4], Jung Sun Yum[2], Sang-Jun Ha[1]

[1]Department of Biochemistry, College of Life Science & Biotechnology, Yonsei University, Seoul 120749, Republic of Korea

[2]R&D Center, CHA Vaccine Institute, Seongnam-si, Gyeonggi 462716, Republic of Korea [3]Jeonbuk Department of Inhalation Research, Korea Institute of Toxicology, Jeollabuk-do 56212, Republic of Korea

[4]Department of Microbiology, Institute for Immunology and Immunological Diseases, Brain Korea 21 PLUS Project for Medical Science, Yonsei University College of Medicine, Seoul 120749, Republic of Korea

[*]To whom correspondence should be addressed. E-mail: jsyum@chamc.co.kr, sjshin@yuhs.ac, or sjha@yonsei.ac.kr.

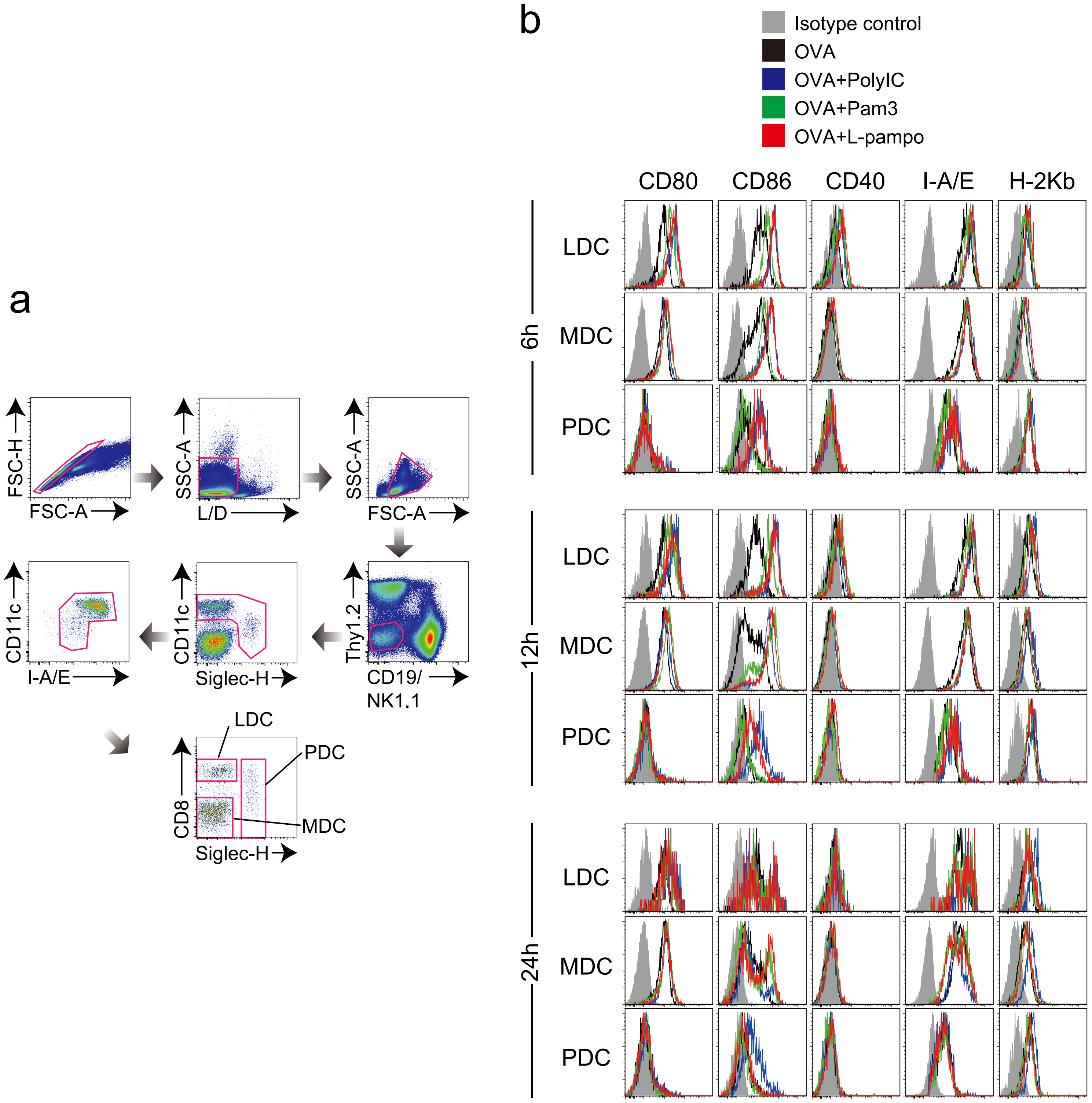

Figure S1. L-pampo does not further enhance the expression of co-stimulatory or MHC molecules on the subtypes of dendritic cells (DCs). Naive B6 mice (*n*=3) were immunized i.p. with 100 μg of OVA alone or in combination with alum, polyI:C, pam3, or L-pampo as adjuvants. After 6, 12, or 24 h, the spleens were dissected and analyzed by flow cytometry. (a) The gating strategy for the subtypes of splenic DCs. (b) Expression of CD80, CD86, CD40, I-A/E, or H-2Kb on the subtypes of splenic DCs. LDC; lymphoid dendritic cell, MDC; myeloid dendritic cell, PDC; plasmacytoid dendritic cell. The data are representative of two independent experiments.

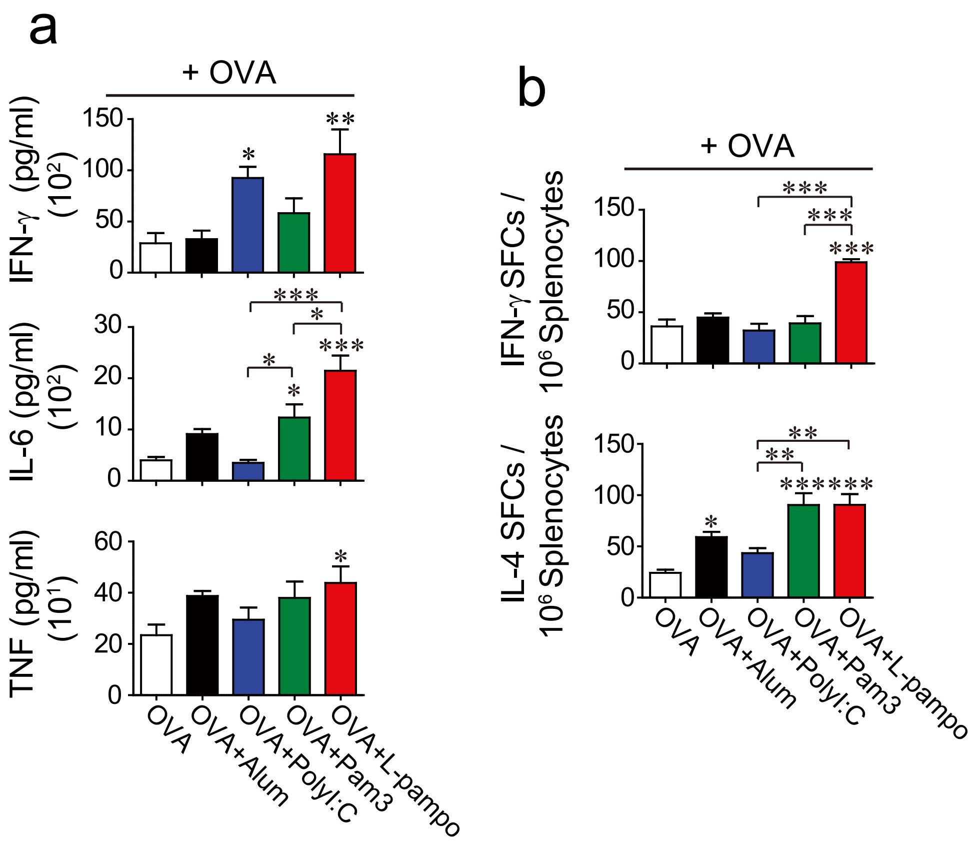

Figure S2. L-pampo enhances multi-cytokine production when T cells are re-stimulated with OVA protein Naïve Ly5.2$^+$ B6/J mice were injected with Ly5.1$^+$ OT-I and OT-II T cells (5 x 10^5 cells each) before immunization, then immunized with 100 µg of OVA alone or in combination with alum, polyI:C, Pam3, or L-pampo as adjuvants, followed by the same immunization 3 and 6 weeks after the first immunization. Seven days after the tertiary immunization, splenocytes were isolated and stimulated *ex vivo* with OVA protein. (a) The levels of IFN-γ, IL-6, and TNF in the supernatants of the re-stimulated splenocytes were measured by a cytokine ELISA. (b) The splenocytes were analyzed for IFN-γ or IL-4 secretion by an ELISpot assay. The data are represented as the mean ± SEM (n=4). *, p<0.05; **, p<0.01; ***, p<0.001.

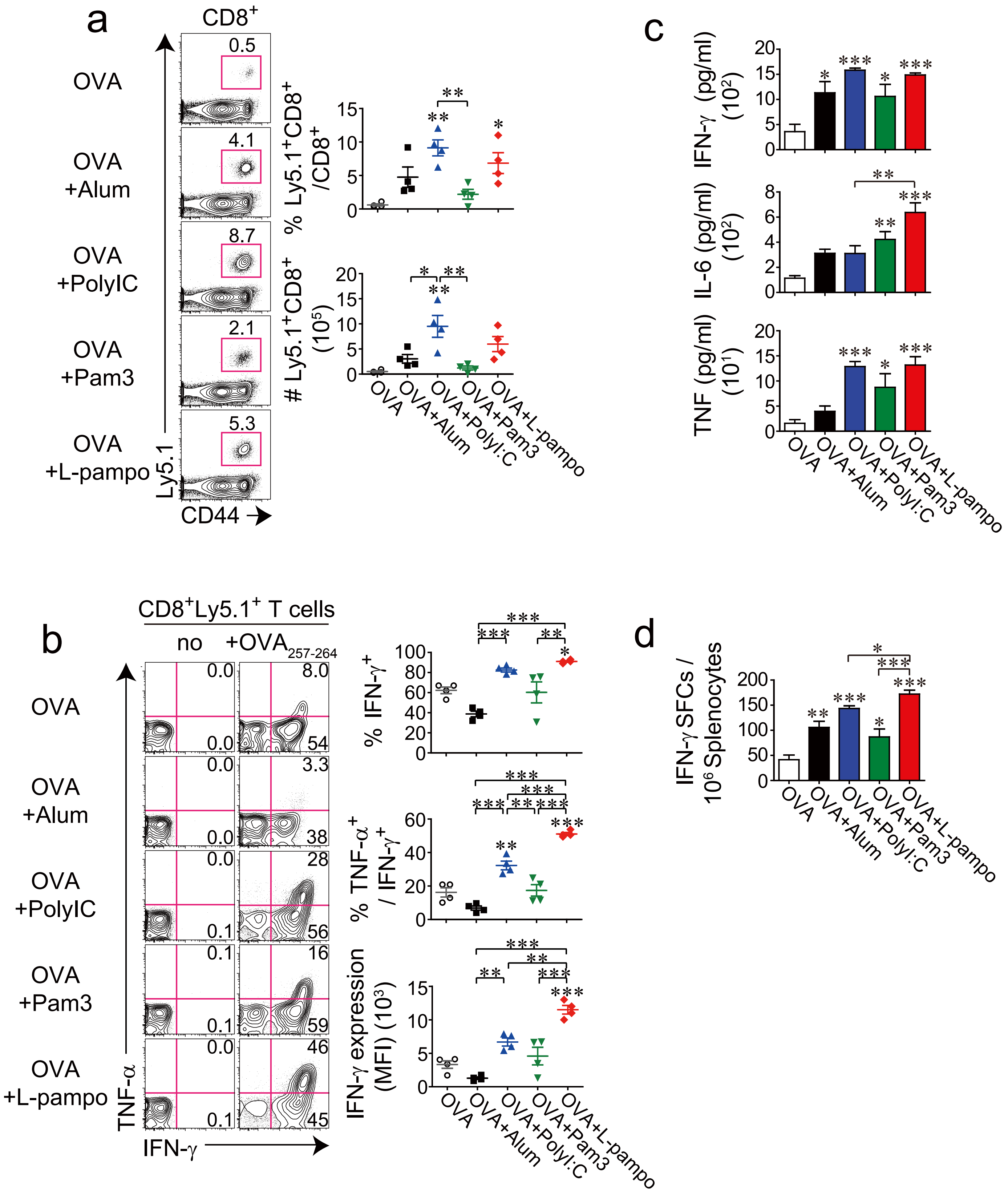

Figure S3. L-pampo enhances the multi-cytokine-producing capability of antigen-specific CD8$^+$ T cells upon boosting. In the same experimental scheme shown in Figure S2, splenocytes were analyzed by flow cytometry. (a) Representative FACS plots and the frequencies and numbers of the transferred OT-I cells (CD4$^+$CD44$^+$Ly5.1$^+$) among CD8$^+$ T cells are shown. (b-d) Splenocytes were stimulated *ex vivo* with OVA$_{257\text{-}264}$ peptides. (b) Representative FACS plots of cytokine-producing OT-I cells and the frequencies of IFN-γ$^+$ cells among the OT-I cells and of IFN-γ$^+$TNF-α$^+$ cells among IFN-γ$^+$ OT-I cells and IFN-γ expression levels of OT-I cells determined by the mean fluorescence intensity (MFI) of IFN-γ are shown. (c) The levels of IFN-γ, IL-6, and TNF in the supernatants of the re-stimulated splenocytes were measured with a cytokine ELISA. (d) The splenocytes were analyzed for IFN-γ secretion with an ELISpot assay. The data are represented as the mean ± SEM with each dot indicating one mouse (*n*=4). *, p<0.05; **, p<0.01; ***, p<0.001.
